# Physical Function in Adults With Metabolic Acidosis and Advanced CKD: Patient Reported Versus Assessed Physical Function

**DOI:** 10.1016/j.xkme.2022.100518

**Published:** 2022-07-07

**Authors:** Navdeep Tangri, Michael Walker, Thomas W. Ferguson, Vandana Mathur

**Affiliations:** 1 Department of Internal Medicine, Max Rady College of Medicine, University of Manitoba, Winnipeg, Manitoba, Canada; 2 Seven Oaks Hospital Chronic Disease Innovation Centre, Winnipeg, Manitoba, Canada; 3 Walker Biosciences, Carlsbad, CA; 4 MathurConsulting Woodside, CA; 5 Clinic by the Bay, San Francisco, CA

To the Editor:

Chronic kidney disease (CKD) is characterized by a progressive reduction in kidney function. In this condition, acid accumulates and is buffered by bone and muscle, leading to bone demineralization and protein catabolism.^1^ Eventually, the increased protein catabolism results in disturbances in skeletal muscle, with reduction in muscle mass and muscle strength, and worsening of physical function. Reduction in physical function begins early in the disease process and is associated with increased mortality risk.^2^^,^^3^

In a recent prospective cohort study, we observed that the time necessary to complete a 5 time sit-to-stand (STS-5) test was associated with all-cause mortality in patients with CKD, and chair stand time declined immediately after dialysis initiation.^4^ However, measurement of physical function using chair stand time is not part of routine clinical assessment or workflow for patients with CKD. As such, the purpose of this study was to assess the correlation between easily obtained survey questions from the 10-item Kidney Disease and Quality of Life Physical Function Domain (KDQOL-PFD) instrument and objectively measured physical performance from the STS-5 to establish the validity of the KDQOL-PFD in assessment of physical function.

Using data from a 1-year phase 3 randomized, placebo-controlled trial in patients with CKD and metabolic acidosis (n = 196), we evaluated the correlation between patient-reported limitation on daily activities as measured by the 10-item KDQOL-PFD scale (also known as the Short Form-36 physical function or SF-36 PF-10 scale) and the STS-5.^5^^,^^6^^,^^7^ Individual items of the KDQOL-PFD are provided in Table S1, and instructions for performing the STS-5 are provided in Item S1. Baseline characteristics of the patients are reported in Table S2. This trial examined the efficacy and safety of veverimer, a nonabsorbed hydrochloric acid binder, as a treatment for metabolic acidosis in patients with estimated glomerular filtration rate 20-40 mL/min/1.73 m^2^ and metabolic acidosis (serum bicarbonate 12-20 mEq/L). In this study, both the STS-5 and the KDQOL-PFD were significantly improved in patients who received veverimer compared to placebo.^5^ Both the STS-5 and KDQOL-PFD were assessed at the same study visits. This multi-site study was conducted across 29 sites with institutional review board or ethics committee approval received at each site. All patients provided written consent before participation in the study, and full results of the trial including patient demographics and clinical characteristics have been previously published.^5^

Correlations between the 2 measures of physical function were calculated using the Pearson product-moment correlation coefficient. In addition, a linear model was fit to determine the association of each category of decline related to activities of daily living (Table S1) in the KDQOL-PFD with the change (measured in seconds) in the STS-5. A clinically important difference in the STS-5 was 1.7 seconds, based on applications in cohorts for other chronic conditions.^8^

In the linear model, each category of decline in the KDQOL-PFD was associated with a statistically significant deterioration in the times from the STS-5 (ranging between 3.29 to 3.80 seconds for each category), all of which were greater than the clinically important difference of 1.7 seconds (Table 1).^8^ There were significant, direct correlations between improvement in the STS-5 time (defined as a short time to complete the test) and the improvement in the KDQOL-PFD (defined as a higher score) over 1 year, with a Pearson product-moment correlation of +0.223, *P* = 0.002). A visual representation of this relationship is provided in Fig 1. In addition, 5 of the 10 KDQOL-PFD items were significantly correlated with improvement in the repeated chair stand time: lifting or carrying groceries; bending, kneeling, or stooping; walking several blocks; walking 1 block; and bathing or dressing oneself (Table 2).

**Table 1:** Linear Models of Change in Repeated Chair Stand Test Time Versus Change in KDQOL-PFD

2.1 Model of Change in Repeated Chair Stand Test Time Vs Change in KDQOL-PFD Total Score				
	Estimate	Std. Error	t-value	*P*
Intercept	−2.39855	0.90961	−2.637	0.00907
KDQOL change	−0.12233	0.03912	−3.127	0.00205

2.2 Model of Change in Repeated Chair Stand Test Time Vs Change in KDQOL-PFD Item C Lifting or Carrying Groceries				
	Estimate	Std. Error	t-value	*P*
Intercept	−2.4599	0.9046	−2.719	0.00716
KDQOL C change	−3.8282	1.2254	−3.124	0.00207

2.3 Model of Change in Repeated Chair Stand Test Time Vs Change in KDQOL-PFD Item F Bending, Kneeling, or Stooping				
	Estimate	Std. Error	t-value	*P*
Intercept	−2.2633	0.9363	−2.417	0.01660
KDQOL F change	−3.2910	1.1654	−2.824	0.00526

2.4 Model of Change in Repeated Chair Stand Test Time Vs Change in KDQOL-PFD Item H Walking Several Blocks				
	Estimate	Std. Error	t-value	*P*
Intercept	**−**2.3768	0.9221	−2.578	0.01072
KDQOL H change	−3.1847	1.1161	−2.853	0.00481

2.5 Model of Change in Repeated Chair Stand Test Time Vs Change in KDQOL-PFD Item I Walking One Block				
	Estimate	Std. Error	t-value	*P*
Intercept	−3.0034	0.8879	−3.383	0.000874
KDQOL I change	−3.4726	1.4695	−2.363	0.019147

2.6 Model of Change in Repeated Chair Stand Test Time Vs Change in KDQOL-PFD Item J Bathing or Dressing Yourself				
	Estimate	Std. Error	t-value	*P*
Intercept	−2.8612	0.8977	−3.187	0.00168
KDQOL J change	−3.6332	1.6492	−2.203	0.02882

Abbreviations: KDQOL, Kidney Disease and Quality of Life; KDQOL-PFD, Kidney Disease and Quality of Life Physical Function Domain.

**Figure 1:**
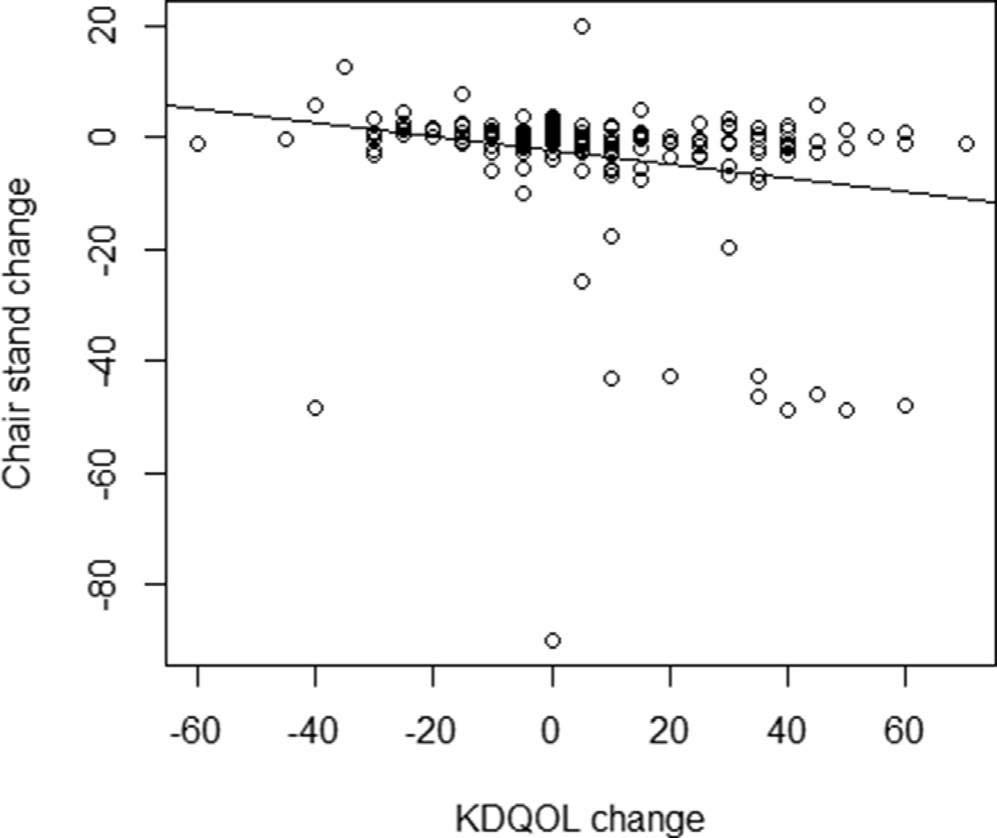
Change in repeated chair stand test time versus change in KDQOL-PFD. Chair stand change measured in seconds; KDQOL change is the difference in the KDQOL-PFD score over 1 year. Abbreviations: KDQOL, Kidney Disease and Quality of Life; KDQOL-PFD, Kidney Disease and Quality of Life Physical Function Domain.

**Table 2:** Improvement (ie, Higher Score) on the KDQOL-PFD Total and Individual Scores Correlated Significantly with Improvement (ie, Faster Time) on the Repeated Chair Stand Test

Variables	Correlation Coefficient (*r*)	95% CI	*P*
Change in KDQOL-PFD total score	+0.223	0.083-0.354	0.002
Change in Individual KDQOL-PFD Item Scores			
Lifting or carrying groceries	+0.223	0.083-0.354	0.002
Bending, kneeling, or stooping	+0.202	0.061-0.335	0.005
Walking several blocks	+0.204	0.063-0.337	0.005
Walking one block	+0.170	0.028-0.306	0.02
Bathing or dressing yourself	+0.159	0.017-0.295	0.03

Abbreviations: CI, confidence interval; KDQOL-PFD, Kidney Disease and Quality of Life Physical Function Domain.

These findings suggest that incorporating the KDQOL-PFD physical function questionnaire as part of the clinical visit workflow for patients with CKD can identify patients with objective impairment in physical function. This short questionnaire that comprises 10 items and requires less than 5 minutes to complete may be particularly useful for patients unable to do the STS-5 and in situations where incorporation of the physical performance measure (chair stand) is infeasible in a particular clinic setting. Integration of this questionnaire in the clinic may provide a rapid and practical mechanism for both identifying at-risk patients as well as quantifying changes in physical function over time. A 3-5 point decrease in the total score of the KDQOL-PFD (score range 0-100) represents a clinically meaningful decline.^9^

Physical performance has important implications for overall health outcomes.^10^ Moreover, the KDQOL-PFD specifically evaluates limitations in activities that affect independent living and risk of injury, if impaired, such as walking, climbing stairs, and bending or stooping. The routine assessment of physical function in this manner can complement the STS-5 and would allow for appropriate interventions to help prevent further impairment and downstream disability and hospitalizations.

## Supplementary Material

Supplementary File (PDF) Item S1, Tables S1-S2.
