# Supplementary material for: Physical Function in Adults With Metabolic Acidosis and Advanced CKD: Patient Reported Versus Assessed Physical Function
Source: Kidney Med. 2022 Jul 7;4(9):100518. doi: 10.1016/j.xkme.2022.100518 (PMC9485586; doi:10.1016/j.xkme.2022.100518)
Supplement: Supplementary File (PDF) — Item S1, Tables S1-S2. [file mmc1.pdf]

Supplemental Table S1: Kidney Disease and Quality of Life – Physical Function Domain

3. The following items are about activities you might do during a typical day. Does your health now limit you in the activities? If so, how much? [Mark an □in a box on each line.]		Yes, limited a lot ▼	Yes, limited a little ▼	No, not limited at all ▼
a	Vigorous activities, such as running, lifting heavy objects, participating in strenuous sports	□	□	□
b	Moderate activities, such as moving a table, pushing a vacuum cleaner, bowling or playing golf.	□	□	□
c	Lifting or carrying groceries	□	□	□
d	Climbing several flights of stairs	□	□	□
e	Climbing one flight of stairs	□	□	□
f	Bending, kneeling, or stooping	□	□	□
g	Walking more than a mile	□	□	□
h	Walking several blocks	□	□	□
i	Walking one block	□	□	□
J	Bathing or dressing yourself	□	□	□

Item S1. Instructions for Performing the STS-5

Required Equipment

- A chair without cushions that has a straight back without arm rests. The following chair dimensions (or close to these) are recommended: seat 44.5 cm (17 ½ inches) high and 38 cm (15 inches) deep.
- A Stopwatch.

Instructions

Instructions to the subject are shown in ***bold italic*** and should be given exactly as they are written in this script. The subject must be able to stand unassisted without the use of a cane or walker.

> ***Now let's begin the repeated chair stand evaluation. I will first describe and show the movement to you. Then I'd like you to try to do it. If you cannot do sit-stand movement, or if you feel it would be unsafe to try to do it, please tell me. Let me emphasize that I do not want you to try to do this exercise if you feel it might be unsafe.***
> ***Do you have any questions before we begin?***

Single Chair Stand (Practice Test)

1. ***Do you think it would be safe for you to try to stand up from a chair without using your arms?***
2. ***This test measures the strength in your legs***
3. (Demonstrate and explain the procedure.) ***First, fold your arms across your chest and sit so that your feet are on the floor; then stand up keeping your arms folded across your chest***
4. ***Please stand up keeping your arms folded across your chest.*** (Record result).
5. If subject cannot rise without using arms, say: ***"Okay, try to stand up using your arms."*** This is the end of their test. Record result and go to the scoring page.

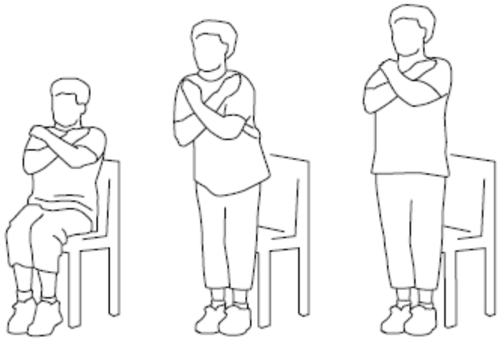

Repeated Chair Stand Test

1. ***Do you think it would be safe for you to try to stand up from a chair five times without using your arms?***
2. (Demonstrate and explain the procedure.) ***Please stand up straight as QUICKLY as you can five times, without stopping in between. After standing up each time, sit down and then stand up again. Keep your arms folded across your chest. I will be timing you with a stopwatch.***
3. When the subject is properly seated, say: ***"Ready? Stand"***, and begin timing using a stopwatch.
4. Count out loud as the subject arises each time, up to five times.
5. Stop if subject becomes tired or short of breath during repeated chair stands.
6. Stop the stopwatch when subject has straightened up completely for the fifth time.
7. Also stop:
   - If subject uses his/her arms
   - After 1 minute, if subject has not completed five rises
   - At your discretion, if concerned for subject's safety
8. If the subject stops and appears to be fatigued before completing the five stands, confirm this by asking: ***"Can you continue?"***
9. If subject says "Yes", continue timing. If subject says "No", stop and reset the stopwatch.

SCORING

Single Chair Stand

		YES	NO
A.	Safe to stand without help	☐	☐
B.	Results:		
	Subject stood without using arms	☐	→ Go to Repeated Chair Stand Test
	Subject used arms to stand	☐	→ End test; score as 0 points
	Test not completed	☐	→ End test; score as 0 points
C.	If subject did not attempt test or failed, circle why:		
	Tried but unable	1	
	Subject could not stand unassisted	2	
	Not attempted, you felt unsafe	3	
	Not attempted, subject felt unsafe	4	
	Subject unable to understand instructions	5	
	Other (Specify)____	6	
	Subject refused	7	

Repeated Chair Stand Test

		YES	NO
A.	Safe to stand five times	☐	☐
B.	If five stands done successfully, record time in seconds.		
	Time to complete five stands __ __ . __ __ seconds		
C.	If subject did not attempt test or failed, circle why:		
	Tried but unable	1	
	Subject could not stand unassisted	2	
	Not attempted, you (person administering test) felt unsafe	3	
	Not attempted, subject felt unsafe	4	
	Subject unable to understand instructions	5	
	Other (Specify)____	6	
	Subject refused	7	

Scoring the Repeated Chair Stand Test

Subject unable to complete 5 chair stands or completes stands in >60 sec:	☐ 0 points
If chair stand time is 16.70 sec or more:	☐ 1 point
If chair stand time is 13.70 to 16.69 sec:	☐ 2 points
If chair stand time is 11.20 to 13.69 sec:	☐ 3 points
If chair stand time is 11.19 sec or less:	☐ 4 points

Supplement Table S2. Baseline characteristics of enrolled patients

	Veverimer (n	
Age (years)	62·9 (12·1)	61·7 (11·9)
≥65	58 (51%)	38 (46%)
Sex		
Male	68 (60%)	51 (62%)
Female	46 (40%)	31 (38%)
Race		
White	113 (99%)	79 (96%)
Black or African American	1 (1%)	3 (4%)
Region		
Europe	108 (95%)	71 (87%)
USA	6 (5%)	11 (13%)
Body-mass index (kg/m²)	28·6 (4·0)	27·9 (3·9)
Systolic blood pressure (mm Hg)	135·9 (8·9)	136·5 (9·0)
Selected medical history		
Hypertension	110 (96%)	79 (96%)
Diabetes	70 (61%)	57 (70%)
Dyslipidaemia, hyperlipidaemia, or hypercholesterolaemia	70 (61%)	48 (59%)
Left ventricular hypertrophy	56 (49%)	35 (43%)
Congestive heart failure	34 (30%)	28 (34%)
Percutaneous coronary intervention or coronary bypass graft	19 (17%)	14 (17%)
Myocardial infarction	17 (15%)	10 (12%)
Stroke	8 (7%)	8 (10%)
Atrial fibrillation or atrial flutter	7 (6%)	6 (7%)
Peripheral vascular disease	5 (4%)	6 (7%)
Peripheral vascular disease intervention or surgical arterial bypass	1 (1%)	3 (4%)
Transient ischaemic attack	1 (1%)	0
Cause of chronic kidney disease		
Hypertension	36 (32%)	27 (33%)
Diabetes and hypertension	37 (32%)	23 (28%)
Diabetes	15 (13%)	18 (22%)
Glomerulonephritis	7 (6%)	7 (9%)
Interstitial nephritis	6 (5%)	3 (4%)
Cystic renal disease	5 (4%)	3 (4%)
Other, unknown, or urological	8 (7%)	1 (1%)
Medication use		
Sodium bicarbonate	10 (9%)	5 (6%)
ACE inhibitors or ARBs	74 (65%)	66 (80%)
Diuretics	70 (61%)	52 (63%)
Calcium channel blockers	65 (57%)	49 (60%)
Anti-diabetic drugs	62 (54%)	45 (55%)
β blockers	53 (46%)	46 (56%)
Lipid modifying agents	49 (43%)	41 (50%)
Anti-thrombotic agents	44 (39%)	41 (50%)

(Table 1 continues in next column)

	Veverimer (n=114)	Placebo (n=82)
(Continued from previous column)		
Laboratory values		
Serum bicarbonate (mmol/L)	17·2 (1·4)	17·1 (1·5)
>18	37 (32%)	23 (28%)
≤18	77 (68%)	59 (72%)
Venous blood pH	7·30 (0·08)	7·30 (0·09)
Venous blood base excess (mmol/L)	−9·3 (2·2)	−9·3 (2·2)
Estimated GFR (mL/min per 1·73 m²)	29·4 (6·4)	27·9 (5·4)
Blood urea nitrogen (mmol/L)	13·7 (4·8)	13·6 (4·6)
Serum creatinine (µmol/L)	193·4 (47·5)	204·3 (51·8)
Serum sodium (mmol/L)	139·9 (2·6)	139·4 (2·9)
Serum potassium (mmol/L)	4·9 (0·6)	4·9 (0·6)
Serum chloride (mmol/L)	106·9 (3·7)	107·3 (4·8)
Serum calcium (mmol/L)	2·3 (0·1)	2·3 (0·1)
Serum phosphate (mmol/L)	1·2 (0·2)	1·2 (0·2)
Serum magnesium (mmol/L)	0·87 (0·11)	0·86 (0·11)
Serum anion gap (mmol/L)*	20·8 (3·9)	20·0 (4·1)
Haemoglobin (g/L)	125·7 (17·7)	125·6 (17·4)
Urine albumin-to-creatinine ratio (mg/mmol)†	23·5 (16·6–33·6)	34·5 (23·4–50·7)
Physical functioning		
KDQoL-PFD total score‡	52·6 (22·4)	55·7 (26·2)
Repeated chair stand (s)§	21·7 (16·9)	21·0 (17·1)

Data are mean (SD), n (%), or geometric mean (95% CI). Baseline characteristics were measured before randomisation in the parent study. ACE=angiotensin converting enzyme. ARBs=angiotensin II receptor blockers. GFR=glomerular filtration rate. KDQoL-PFD=Kidney Disease and Quality of Life-Physical Function Domain. *Serum anion gap was calculated as sodium plus potassium minus (chloride plus bicarbonate). †Values are from a spot urine collection. ‡Veverimer n=114; placebo n=82. §Veverimer n=114; placebo n=81.

Table 1: **Baseline characteristics of all enrolled participants**

Reprinted from Wesson et al(5) with permission of Elsevier.
